# NIA DIVISION OF NEUROSCIENCE

**DOI:** 10.1093/geroni/igad104.0836

**Published:** 2023-12-21

**Authors:** Jennie Larkin

**Affiliations:** National Institute on Aging, Bethesda, Maryland, United States

## Abstract

Dr. Larkin will discuss research priorities for the NIA Division of Neuroscience. Dr. Larkin will also be available for small group discussion.

